# Grammatical Immune System Evolution for Reverse Engineering Nonlinear Dynamic Bayesian Models

**DOI:** 10.4137/cin.s694

**Published:** 2008-08-28

**Authors:** B.A. McKinney, D. Tian

**Affiliations:** Department of Genetics, University of Alabama School of Medicine, Birmingham, AL 35294

**Keywords:** artificial immune system, somatic hypermutation, V(D)J recombination, unscented kalman filter, dynamic bayesian network, estrogen metabolism, nonlinear dynamic bayesian model

## Abstract

An artificial immune system algorithm is introduced in which nonlinear dynamic models are evolved to fit time series of interacting biomolecules. This grammar-based machine learning method learns the structure and parameters of the underlying dynamic model. *In silico* immunogenetic mechanisms for the generation of model-structure diversity are implemented with the aid of a grammar, which also enforces semantic constraints of the evolved models. The grammar acts as a DNA repair polymerase that can identify recombination and hypermutation signals in the antibody (model) genome. These signals contain information interpretable by the grammar to maintain model context. Grammatical Immune System Evolution (GISE) is applied to a nonlinear system identification problem in which a generalized (nonlinear) dynamic Bayesian model is evolved to fit biologically motivated artificial time-series data. From experimental data, we use GISE to infer an improved kinetic model for the oxidative metabolism of 17*β*-estradiol (E_2_), the parent hormone of the estrogen metabolism pathway.

## 1. Introduction

The goal of systems biology is to understand the network of interacting genes, proteins, and biochemical reactions that regulate systemic properties of an organism. A realistic biological network, rather than a static graph, should contain nodes that produce time-varying input/output and edges that represent flux through the system [1]. For many biological pathways there is a lack of accurate mathematical models capable of capturing causal dependencies and mechanistic information contained in kinetic data. Thus, one of the goals of computational biology is to develop data-driven algorithms to automate the identification of mathematical model structure from time series. Dynamic Bayesian networks (DBNs) have been used to identify linear relationships between variables in gene networks from time series [2, 3, 4]. However, in addition to time-dependence, a realistic biological network should capture nonlinear relationships. We use a flexible differential equation formalism that allows us to treat time-dependent nonlinearities in the form of a nonlinear dynamic Bayesian model (NDBM).

Specifically, we use a Kalman Filter (KF) to optimize model parameters and determine the accuracy of models that track time series. The KF is a tracking and estimating tool widely used in engineering and has been applied to the inference of NDBMs from biological time series [7]. The KF is a Bayesian method in the sense that it provides a mechanism to incorporate prior information from a previous time point to update the state of the system at the current time point. For linear models, the KF reduces to a DBN; however, the KF becomes the more general NDBM when a nonlinear model is specified. Although the KF provides an efficient, recursive method for identifying the parameters of a NDBM, the enormity of the search space of possible nonlinear model structures calls for heuristic search methods, such as evolutionary algorithms [5, 6, 7], to identify the underlying structure. An important challenge in time-series bioinformatics addressed in this work is the automated, data-driven identification of mathematical model structures.

Previously we implemented a hybrid grammatical evolution (GE) approach to infer nonlinear model structures and parameters from time series [7]. The advantage of GE lies in the simplicity of translating variable-length binary string genomes into programs using a context-free grammar [8]. However, the advantage of the context-free grammar can lead to problems during crossover and mutation because model segments downstream of a mutation or crossover-point that possessed an evolutionary advantage in the parent chromosome may have a completely different meaning in the context of the offspring. Thus, there are theoretical reasons that crossover and mutation can be destructive operators in GE [9]. Novel crossover methods, such as homologous crossover [10] and tree-based crossover [11], which attempt to preserve model building blocks, typically show no improvement over standard GE crossover techniques. In the application domain of human genetics, it has been shown that the performance of GE was not statistically different from a random search [12].

In Sections 1–4 we introduce a new grammar-based, artificial immune system algorithm called Grammatical Immune System Evolution (GISE). To overcome the destructive nature of evolutionary operators in other grammar-based evolutionary algorithms, we introduce a hypermutation operator that preserves evolutionarily fit model features. As we discuss in more detail in Sec. 2, we focus on hypermutation because of its important biological role in secreting high-affinity antibodies with increased antiviral function. GISE takes advantage of a grammar’s ability to restrict the search space based on domain knowledge, while at the same time limiting the damage caused by evolutionary operators. As discussed in Secs. 3 and 4, when generating programs, the grammar inserts nonterminal information into an untranscribed pre-program, and this information is excised after transcription and subsequent expression of the antibody (i.e. the dynamic model). Rather than acting on GE binary strings or directly on GP programs, GISE evolutionary operators act on these intermediate pre-programs. Mutation is initiated by a break in the untranscribed pre-program. The grammar then acts as an error-prone DNA repair mechanism using the nonterminal information previously inserted by the grammar as a repair signal. These nonterminal repair signals enforce semantic constraints imposed by the grammar, thereby preserving evolutionarily fit model segments.

We use GISE to automatically reverse engineer NDBMs to fit time-series data simulated to include nonlinearity and interactions (Secs. 5.1 and 5.2). We demonstrate that GISE, unlike GE, greatly outperforms a Monte Carlo search for realistic nonlinear simulated models. In Sec. 5.3 we apply GISE to experimental time-series data for the oxidative metabolism of 17*β*-estradiol (E_2_). We infer an improved model of the kinetics of this pathway that has been implicated in breast cancer. In this application of the immune system heuristic, the time series represents the antigen, the candidate models represent antibodies, and the goodness of fit as determined by the KF is analogous to the binding affinity.

## 2. Artificial Immune Systems

Despite having an estimated genome of fewer than 25,000 protein-encoding genes, the human adaptive immune system is able to recognize tens of millions of antigens. This seemingly limitless ability to generate diveristy has inspired the development of evolutionary algorithms that mimick features of the adaptive immune sytem. These algorithms are known as artificial immune systems [13], and they typically utilize high-level features of the immune system such as clonal selection, negative selection, and immunological memory. With the aid of a grammar, we attempt to simulate the molecular-level immunogenetic mechanisms that generate diversity in antigen receptors, such as immunoglobulin in B cells and the T cell receptor (TCR) in T cells.

The ability to create such a diverse array of antigen-specific antibodies from a finite set of genes from the germline is accomplished primarily through a genetic reshuffling process known as V(D)J recombination and a diversification process known as immunoglobulin hypermutation (IHM). However, there is experimental evidence to suggest that somatic mutation is the key to creating high-affinity antibodies. It is well known that virus-induced antibodies in infants exhibit poor functional activity compared to that of adults. For example in rotavirus it was shown that, although infant antibody gene sequences use the same immunodominant gene segments as adults to respond to the virus, there was a marked lack of somatic mutations in the infant antibody sequences [14]. Recently we found that human adult antibodies specific to rotavirus bind in a region enhanced by somatic mutations and these mutations account for the enhanced affinity of the adult antibodies [15, 16]. Thus, the grammar-based immune system algorithm described in this work focuses on creating a computational model of the immunogenetic mechanism of hypermutation for generating dynamic model diversity because of the important biological role of IHM in the secretion of high-affinity antibodies with increased antiviral function.

## 3. Grammatical Immunoglobulin Hypermutation

The mechanism of IHM is not completely understood. IHM has been linked to transcription and requires the presence of immunoglobulin enhancers [17]. In addition to random nuculeotide substitutions, certain hotspot motifs have an increased susceptibility to mutation [18]. Mutations are introduced into the antibody genes by an error-prone polymerase during the repair of double stranded breaks (DSBs). The resulting antibody generally preserves the antibody architecture established by V(D)J recombination. It has been shown that hypermutation can occur in any antibody sequence provided there is an active promoter and immunoglobulin enhancer. Promoters and enhancers are types of regulatory regions that help control the transcription of genes. Enhancer DNA sequences bind transcription factors called enhancer-binding proteins that increase the rate of transcription. In the grammar-based hypermutation operator used by GISE, the grammar plays the role of the DSB repair mechanism and nonterminal tags are used as promoters and enhancers.

**Figure 1:**
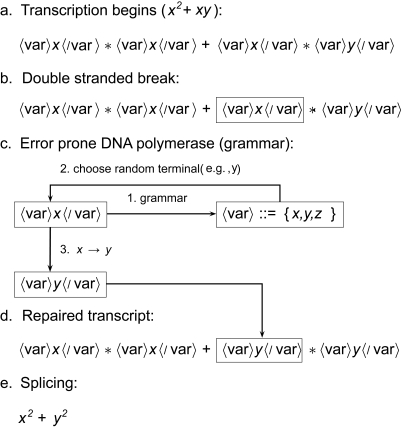
Illustration of grammatical hypermutation (GHM) used in grammatical immune system evolution (GISE). GHM is applied to an untranscribed program corresponding to *x*^2^ + *xy* (**a**). A double stranded break occurs (**b**. hatched), and the error-proned grammar repairs the program (**c**). The terminal *x* is replaced by *y* (**d**), and the final spliced program becomes *x*^2^ + *y*^2^ (**e**).

The steps of Grammatical Hypermutation (GHM) employed by GISE are illustrated in Figure 1, in which an initial program *x*^2^ + *xy* leads to the final mutated program *x*^2^ + *y*^2^. A grammar is a set of production rules that can produce sentences in any language. Sentences created by our grammar are systems of coupled nonlinear differential equations. We use a formal notation for describing the syntax of a context-free grammar as a set of production rules that consist of terminals (model elements) and nonterminals (the production rules themselves) [7, 8, 19]. For simplicity, only (var) nonterminals are used in the Figure 1 illustration. In order to preserve the model architecture, the grammar creates an “untranscribed” model with explicit nonterminal elements (Step **a**). In the untranscribed program, terminals are enclosed by nonterminal tags (e.g. 〈var〉 · 〈/var〉). These nonterminal tags are similar to enhancers that will be spliced out after transcription. In Step **b**, a DSB occurs at a random location (hatched region), which specifies the nonterminal of the model that will be mutated. Error-prone repair is initiated in Step **c**. It is determined from the grammar that there are three possible terminals for the (var) enhancer, which are {*x*, *y*, *z*}, and from these terminals *y* is chosen by chance to replace *x*. The replacement is carried out in Step **d**. The grammar tags of the mutated model are spliced out in Step **e**, and the expressed antibody (mathematical model) is introduced to the antigen (i.e. the program fitness is evaluated on the data).

The nonterminal tags in the untranscribed program allow the grammar to interpret information from the enhancer and maintain context within the model. This helps GHM prevent the destruction of good model elements downstream of the DSB. This is especially important during model refinement. For example, let us assume the target model in Figure 1 is, indeed, *x*^2^ + *y*^2^. Whereas GHM in GISE will not modify the *y* terminal from the original model downstream of the targeted mutation *x* (Fig. 1b), there is a finite probability that GE would mutate the final terminal in addition to the terminal targeted for mutation. The context-free nature of the grammar causes downstream GE program elements to be sensitive to small changes in the mutated element; small changes that may lead GE down a very different grammar path. In addition, the degree of context-free sensitivity will depend on the complexity of the grammar. Through the enhancer tags (e.g. 〈var〉 · 〈/var〉), GISE essentially adds context to the context-free grammar by forcing models to respect the constraints of the model prior to the action of the GHM operator.

## 4. Generalized Dynamic Bayesian Network

**Figure 2:**
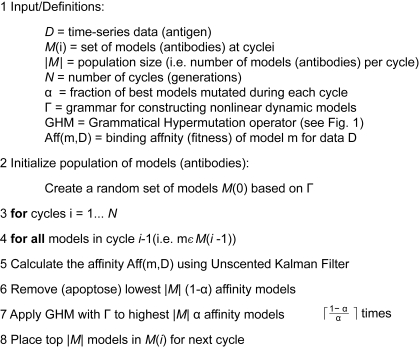
Pseudocode for Grammatical Immune System Evolutionary algorithm applied to the learning of nonlinear dynamic Bayesian models from time series.

Hypermutation in Figure 1 is the major operator of the full GISE algorithm, illustrated as pseudocode in Figure 2. GISE evolves a population of NDBM models of population size |*M*| for *N* cycles of hypermutation and selection. The population of models at cycle *i* is given by *M*(*i*), where each model in the GISE analogy represents an antibody encoded by a plasma B cell. For cycle 0 (line 2) an initial population of models *M*(0) is generated at random from the user-specified grammar Γ. These models represent the germline set of antibodies that will be somatically hypermutated for cycles 1… *N* (line 3). The user specifies the fraction α of models that are hypermutated at each cycle *i* with the help of the grammar Γ During a cycle, the binding affinity (Aff(m,D), line 5) is calculated for all models m with respect to the data D. The time-series D acts as the antigen in Aff(m,D), and the ability of the model (antibody) to fit (bind to) the data (the antigen) is computed using the KF discussed below. In line 6, the models with the lowest affinity for the data die by apoptosis and are removed from the population. Then in line 7, hypermutated models are generated from the highest affinity models to repopulate the set of models. Thus, in line 8, the population is filled with the best models (the highest affinity models and their hypermutated models), which become the basis for the next cycle of selection and hypermutation beginning at line 3. After *N* cycles have been completed, the best models are inspected.

The GISE steps in Figure 2 are primarily concerned with learning the structure of the NDBM. Crucial to the success of the algorithm, however, is the estimation of model parameters and the calculation of goodness-of-fit (affinity, Aff(m,D)) of the model m for the data D, which we carry out using the Unscented Kalman Filter (UKF). We use a discrete nonlinear deterministic state space model for the state at observed time point *t_k_* _+ 1_ in terms of its predecessor at time *t_k_*:

(1) $$y_{k+1}=F(y_{k},\lambda_{k})+\eta_{k},$$

and

(2) $$F(y_{k},\lambda_{k})=y_{k}+\int_{t_{k}}^{t_{k+1}}f(y(T),\lambda )dT,$$

where ***f*** satisfies the coupled set of differential equations

(3) $$\dot{y}(t)=f(y(t),\lambda ),$$

and *ẏ* represents the derivative of a molecular quantity with respect to time. The measurement noise is represented by *η_k_* and enters into our calculations implicitly through the covariance matrix *R_k_* = *E*[*η_k_η_k_^T^*], which becomes part of the covariance matrix ***P****_ỹỹ_* calculation below. The operator *E*[·] is the expectation value. The elements of the vector *λ* of dimension *D_λ_* are the parameters of the model ***f***. Since the model parameters are unknown, we treat the elements of *λ* as state variables to be tracked with process noise **ε**:

(4) $$\lambda_{k+1}=\lambda_{k}+{ɛ}_{k}.$$

The process noise in the stochastically varying parameter vector is represented by **ε**, but this noise vector enters into our calculations only indirectly through the covariance matrix *Q_k_* = *E*[**ε***_k_***ε***_k_^T^*]. Equations (1,2,3,4) can be viewed as a representation of a nonlinear dynamic Bayesian model (NDBM). These equations reduce to a DBN when the vector of nonlinear functions ***f*** is replaced by a linear transformation of ***y*** with a matrix ***A*** (i.e. **Ay** + **ε**). Next we describe an optimal, recursive method—the UKF—to estimate the NDBM parameters *λ* and the model fitness Aff(m,D).

**Figure 3:**
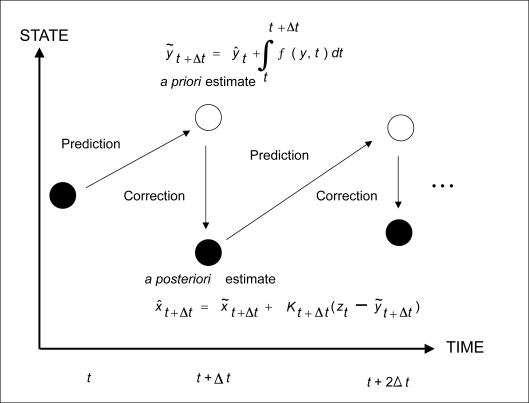
Bayesian concept of the Kalman Filter. Empty circles represent *a priori* state estimates at a given time point before the experimental data is observed and filled circles represent *a posteriori* state estimates that incorporate the experimental data at the given time point. The recursive Kalman Filter steadily improves the estimate of the augmented state, which includes the parameters, as it steps through the observed time points.

The diagram in Figure 3 illustrates the Bayesian estimate of the states of a given model. It is convenient to create an augmented state ***x*** of the system in which the dependent variables ***y*** are augmented by the parameter vector **λ**:

(5) $$x_{k}=\left(\begin{array}{l} \lambda_{k}\\ y_{k} \end{array}\right).$$

Empty circles in the figure represent *a priori* estimates of the states prior to observation of the experimental data *z* at the given time point. Variables with a tilde indicate *a priori* estimates and with a hat indicate *a posteriori* estimates. The *a priori* estimate of the unaugmented variables (e.g. corresponding to observed molecular quantities) at time *t* + Δ*t*, *ỹ_t_*_+Δ_*_t_*, is given by the posterior estimate at the previous time point *t*, *ŷ_t_*, integrated out to *t* + Δ*t*. We use a fourth-order runge-kutta solver to integrate the model Equation 3. In the *a posteriori* estimate of the augmented state *x̂_t_*_+Δ_*_t_* (Eq. 5), we use the Kalman gain matrix *K* to blend the difference between the experimental data *z_t_* and the predicted prior estimate ***ỹ****_t_*_+Δ_*_t_*. In order to make the dimensions agree between the augmented and unaugmented vectors (Fig. 3 and Eq. 6), the Kalman matrix includes a constant contribution Q in the covariance matrix ***P****_x̃ỹ_* =*E*[(***x***−***x̃***)(***y***−***ỹ***)*^T^*]) corresponding to the parameters **λ** in the augmented state. This constant covariance contribution mimics the uncertainty of the variables being tracked. The larger the assumed value of Q, the more relaxed the search for the optimal parameters. The recursive engine of the Kalman filter involves correcting the predicted moments at time point *k* + 1 with the observed data using equations

(6) $${\hat{x}}_{k+1|k+1}={\tilde{x}}_{k+1|k}+K_{k+1}(z_{k}-{\tilde{y}}_{k+1|k})$$

and

(7) $$K_{k+1}=P_{\tilde{x}\tilde{y}}P_{\tilde{y}\tilde{y}}^{-1}.$$

For convenience of notation, we use *k* as the time index, so that if the system is at time *t*, then the current state is ***x****_k_* and the state at time *t* + Δ*t* is ***x****_k_* _+ 1_. The *a posteriori* estimate of the augmented state at time step *k* + 1, given by Equation (6), consists of the *a priori* prediction ***x̃****_k+_*_1|_*_k_* at the previous time step and a correction term proportional to the difference between the observed data *z_k_* and the estimate of the unaugmented state ***ỹ****_k+_*_1|_*_k_* at the previous step. In Equation (7), ***P****_x̃ỹ_* is the covariance matrix for the deviation of the *x* and *y* states from their *a priori* estimates. The Kalman gain or blend matrix ***K***, updated by Equation (7), is chosen to minimize the trace of the *a posteriori* error covariance matrix ***P****_x̂x̂_* because the trace of this covariance matrix equals the sum of the squared errors of the components of the posterior estimate of ***x*** (i.e. *x̃_k+_*_1|_*_k_*_+1_).

We use the unscented transformation (UT) [20] to efficiently and accurately estimate the first two moments of the state distribution undergoing transformations during the prediction of the future state of the system and during the correction of the state with the observation model. The UT estimate of the posterior mean and covariance is accurate to third order for any nonlinearity, and the UKF is much faster than Monte Carlo Markov chain particle filters. Further details on the unscented transformation for the deterministic calculation of the statistics of a random variable undergoing a nonlinear transformation and the application to Kalman filtering for state space modeling can be found in Refs. [7, 20, 21].

## 5. Theoretical and Experimental Validation of GISE

Testing novel system identification algorithms on real biological data is challenging because the true causal connections underlying the system often are unknown. Thus, before applying GISE to real experimental data, we apply it to simulated data from two biological pathway targets for method validation. We simulate 50 time points for each varying quantity ***y***, and we assume *Q* = 0.015 process noise for estimation of parameters λ. When the UKF algorithm is called by GISE, all model parameters are initialized to 0.001. This corresponds to an uncoupled system of differential equations, which is the least biased initialization of the model parameters. An iterative process of parameter optimization is implemented to achieve precise model parameters [7]. The vector of parameter estimates at the final time point are compared with the parameters from the previous iteration, which are used as the input for the next iteration. The UKF process is continued until convergence of the parameters to within a tolerance of 0.0001 is achieved or a maximum loop count of 200 is reached.

To avoid over-fitting, we use the Akaike Information Criterion (AIC) [22] for distinguishing between models ***f***(***y***(*t*), *λ*, **ε**(*t*)). Thus, the model fitness (affinity) is given by:

(8) $$Aff(f)=-2\text{1n}[(f(y,\lambda ,ɛ)|y)]+2D_{\lambda },$$

where ln[*L*] is the maximum log-likelihood and *D***λ** is the complexity of the model, or the number of parameters *λ* in ***f***. AIC avoids over-fitting by balancing the bias of the model predictions with the complexity of the model structure. Once a full population of models has been evaluated, GISE copies the top 20% (the quantity *α* in Fig. 2) of the most fit models to the next cycle. For each of these models, four hypermutated copies are created to fill the remainder of the cycle’s population by GHM, carried out as described in Figure 1.

### 5.1. Application to operon simulations

The first multiline, nonlinear dynamic target pathway is based on the following operon model:

(9) $$\begin{array}{l} {\dot{y}}_{1}=\frac{\kappa_{1}}{\theta_{1}+y_{3}}-\gamma_{1}y_{1},\\ {\dot{y}}_{2}=\kappa_{2}y_{1}-\gamma_{2}y_{2},\\ {\dot{y}}_{3}=\kappa_{3}y_{2}-\gamma_{3}y_{3}, \end{array}$$

where ***k*** = (0.9, 1.0, 0.6), γ = (1.0, 0.6, 0:8), and *θ*_1_ = 0.9 with initial conditions ***y***(0) = (1.0, 0.6, 0.0). First introduced in Ref. [23] and then extended in Ref. [24], the operon model continues to be a useful framework for modeling biological systems [25, 26]. Equation (9) represents a model of an hypothetical single-gene regulatory network involving a negative feedback loop with measured gene products ***y***. A single gene with mRNA concentration *y*_1_ produces an enzyme with concentration *y*_2_. Enzyme *y*_2_ catalyzes a reaction step leading to metabolite *y*_3_, which inhibits the gene that codes for the enzyme. Parameters ***k*** and *γ* are production and degradation constants, respectively, and *θ* modulates the inhibitory Hill function.

The following is the type of grammar used by GISE to evolve a gene regulatory network based on an operon model.

$$\begin{array}{llll} \langle \text{model}-\text{expr}\rangle & ::= & \langle \text{function}\rangle +\langle \text{function}\rangle & \\ \langle \text{function}\rangle & ::= & \langle \text{linear}\rangle & \left(0\right)\\ & | & \langle \text{linear}\rangle & \left(1\right)\\ & | & \langle \text{regulatory}\rangle & \left(2\right)\\ \langle \text{linear}\rangle & ::= & \left(\langle \text{param}\rangle *\langle \text{variable}\rangle \right) & \\ \langle \text{regulatory}\rangle & ::= & \left(\langle \text{param}\rangle /(\langle \text{param}\rangle +\langle \text{variable}\rangle )\right) & \\ \langle \text{variable}\rangle & ::= & y[\langle \text{random}-\text{int}\rangle ] & \\ \langle \text{param}\rangle & ::= & p[\langle \text{incremented}-\text{int}\rangle ] & \end{array}$$

Models are constructed from the grammar beginning with the 〈model-expr〉 nonterminal and subsequent nonterminals are replaced by the grammar rules recursively until the model contains only terminals. Multiple choices for a rule are delimited by a vertical bar. Subtraction operators are not needed because the UKF is able to determine the sign of the model parameters. To manually add parsimony pressure, we add an extra 〈linear〉 to the 〈function〉 choices. When the grammar encounters a 〈variable〉 nonterminal during model construction, 〈random-int〉 assigns a random index to the dependent variable from the possible number of dependent variables. The number of parameters is tracked during model construction so that when a 〈param〉 nonterminal is encountered, 〈incremented-int〉 assigns the next index to the parameter array ***p***.

**Figure 4:**
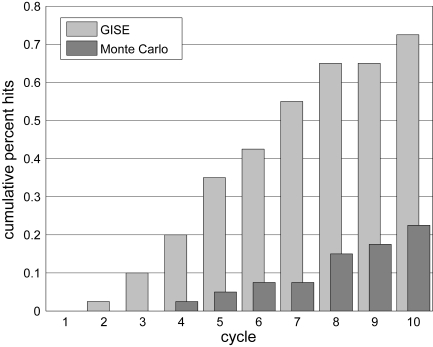
Comparison of Grammatical Immune System Evolution (GISE) and Monte Carlo search to assess whether somatic hypermutation contributes to learning. Monte Carlo is equivalent to GISE without somatic hypermutation, and both execute the same number of function evaluations. Population size is 50. Cumulative percent of runs (out of 100) that identify the correct simulated target (Eq. 9). By cycle 10, 73% of the GISE runs have hit the target versus 23% for Monte Carlo.

Figure 4 shows the cumulative percent of runs (out of 100) that identify the simulated target model of Equation (9) for GISE compared with Monte Carlo search (MCS). Each bar gives the percentage of runs that have identified the correct model at the given cycle. This is an important evaluation of GISE because GE was previously shown to perform no better than a random search for supervised learning in the application domain of human genetics [12]. To make the comparison fair, our MCS constructs models from the same grammar used by GISE. Both algorithms perform the same number of function evaluations (population size = 50), only MCS does not use GHM. Each GISE run takes 4–5 hours to run on a single 3.2 GHz Intel Xeon processor. For all cycles, GISE has a much higher percentage of hits, and by cycle 10, 73% of the GISE runs have hit the target model versus 23% for MCS. This demonstrates the GHM operator in GISE promotes learning by preserving useful model components. We stopped GISE and MCS at a number of cycles sufficient to demonstrate that GISE performs better than MCS for the given model search space. If a large enough number of random models are generated, even MCS will identify the target model, but this type of brute force approach is not practical for higher-dimensional data where more appreciable learning is needed. In practice, the GISE population size and number of cycles should be tuned to account for the size of the search space, or the number of molecular quantities being modeled and for the computational resources available.

### 5.2. Application to S-system simulations

The second, more complex, target model is based on the S-system (synergistic) formalism:

(10) $$\begin{array}{l} {\dot{y}}_{1}=5y_{3}/y_{5}-10y_{1}^{2},\\ {\dot{y}}_{2}=10y_{1}^{2}-10y_{2}^{2},\\ {\dot{y}}_{3}=10/y_{2}-10y_{3}^{2}/y_{2},\\ {\dot{y}}_{4}=8y_{3}^{2}/y_{5}-10y_{4}^{2},\\ {\dot{y}}_{1}=10y_{3}^{2}/-10y_{5}^{2}, \end{array}$$

where the initial conditions are ***y***(0) = (0.7, 0.12, 0.14, 0.16, 0.18). This S-system gene network of five differential equations has been used to test various system identification algorithms [5, 27, 28]. In this two-gene network, *y*_1_ is the mRNA produced from gene 1, *y*_2_ is the enzyme encoded by gene 1, and *y*_3_ is an inducer protein catalyzed by *y*_2_. The quantity *y*_4_ is the mRNA produced from gene 2 and *y*_5_ is a regulator protein encoded by gene 2. Positive feedback from *y*_3_ and negative feedback from *y*_5_ are assumed in the production of mRNAs from the two genes.

The S-system gene network model of Equation 10 is a more realistic test of GISE because of the larger number of variables and because we are using a grammar with opeon-model components instead of biasing the grammar with S-system components used to simulate the gene network based on Equation 10. We use the same operon grammar used in Sec. 5.1 with the exception that we add another rule to the (model-expr) nonterminal in order to more reliably fit this larger system:

$$\begin{array}{llll} \langle \text{model}-\text{expr}\rangle & ::= & \langle \text{function}\rangle +\langle \text{function}\rangle & \left(0\right)\\ & | & \langle \text{function}\rangle +\langle \text{function}\rangle + & \\ & & \langle \text{function}\rangle & \left(1\right) \end{array}$$

**Figure 5:**
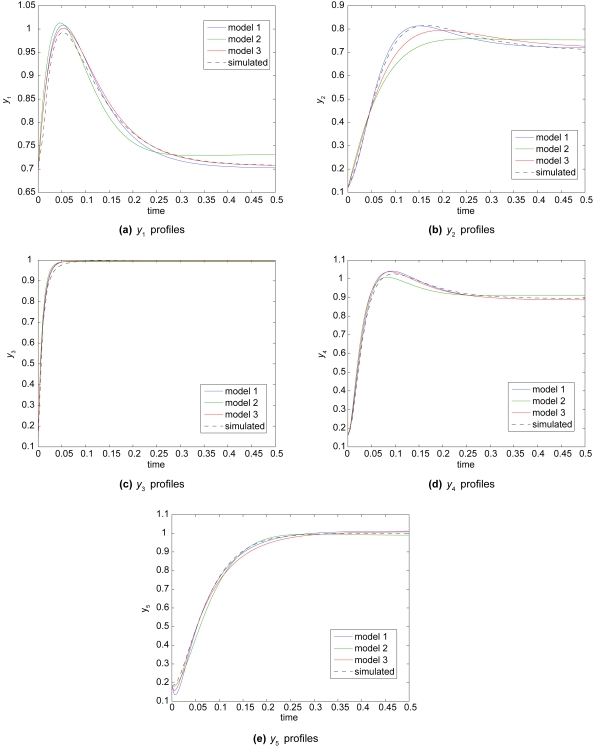
Comparison of top 3 Grammatical Immune System Evolution (GISE) candidate models for the target coupled system simulated by Equation 10 (dashed). GISE population size is 50 and number of cycles is 20. Each subfigure plots the predictions for each *y* profile and the corresponding target profile. (**a**) Model 3 fits *y*_1_ best and has no false positive or false negative connections (see Table 1 and Eq. 10). Model 1 is the next best fit, but it has one false positive connection for *dy*_1_/*dt*. (**b**) Model 1 fits *y*_2_ best. However, it has one false positive connection for *dy*_2_/*dt*, while model 3 has no false positive or negative connections. (**c**) Predictions for *y*_3_ all very similar for all models. (**d**) Predictions by models 1 and 3 are very similar and both have no false positive or negative connections. (**e**) Model 1 fits *y*_5_ slightly better than model 3, but model 1 has one false positive connection.

**Figure 6:**
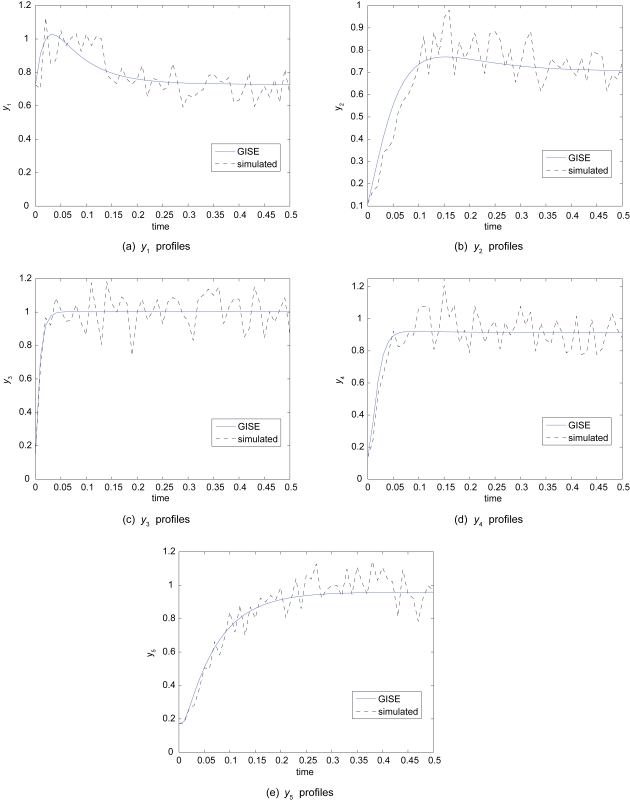
Demonstration of Grammatical Immune System Evolution (GISE) in the presence of noise. GISE models (solid) for the target coupled system simulated by Equation 10 (dashed) corrupted by 10% Gaussian noise.

From Figures 5a–e, we see that GISE is able to fit the S-system simulated data using an operon grammar with reasonable accuracy. A GISE run on this larger data set to find the optimum model structure and parameters takes 7–8 hours to run on a single 3.2 GHz Intel Xeon processor. Each subfigure shows a different y profile for the top 3 GISE model predictions. Even though model 2 has a better goodness of fit, one can see from Figures 5a–e that model 1 fits the data better, possibly over fitting. Model 2 has a higher fitness because it has one fewer parameter than the other two models and hence is more parsimonious. Model 3, with the worst goodness-of-fit of the three top models, may actually be the most desirable model because its causal connections all agree with the simulated model (compare the connections in Model 3 in Table 1 with Eq. 10). In model 1 by contrast, *dy*_2_/*dt* has a false positive connection, *dy*_3_/*dt* has a false negative connection, and *dy*_5_/*dt* has a false positive connection. We define a false positive connection as a model term that predicts a spurious connection between variables, and a false negative connection as a true connection between variables that was not predicted by the model. In Figure 6, we corrupt data simulated by the model given by Equation 10 with 10% Gaussian observation noise and show that the GISE and the UKF can handle the type of noise that often arises in biological data.

### 5.3. Estrogen metabolism pathway

**Figure 7:**
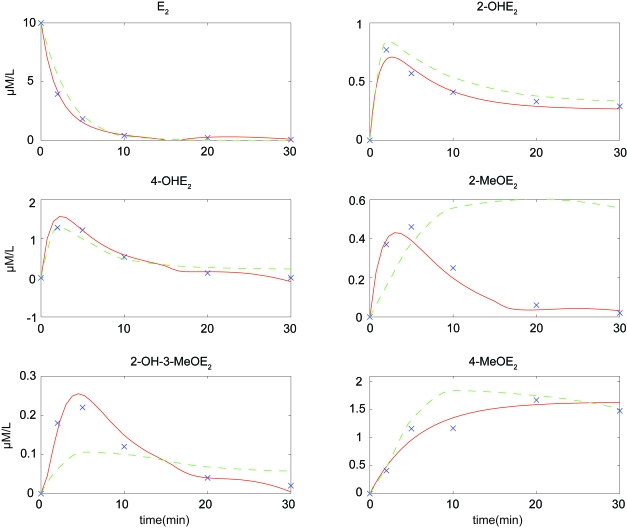
Tracked states for Grammatical Immune System Evolution (GISE) model (*red solid*) of experimental time-series concentrations of estrogen metabolites (*blue x’s*). Concentrations measured in *μ*M/L. GISE model compared with mathematical model derived in Ref. [32] (green dashed).

We now apply GISE to experimental estrogen metabolism time series. Estrogens have been implicated in the development of breast cancer by simultaneously stimulating cell proliferation and gene expression via the estrogen receptor and by causing DNA damage via their oxidative products, the 2-OH and 4-OH catechol estrogens [29, 30]. To better understand estrogen metabolism in the breast, the authors in Ref. [31] employed gas and liquid chromatography with mass spectrometry to measure E_2_, the catechol estrogens 2-hydroxyestradiol (2-OHE_2_) and 4-hydroxyestradiol (4-OHE_2_) as well as methoxyestrogens and estrogenglutathione conjugates. Using this data, a mathematical model was constructed in Ref. [32] using quasi steady-state assumptions and experimental rate constants. To test the computational method described in the current paper, we employed GISE to learn a nonlinear model structure and model parameters directly from the phase-I estrogen time series. The resulting GISE model (Eq. 11) showed an improved fit to the data (Fig. 7) over the previously constructed model in Ref. [32]. Regarding causal connections, there was no significant change in the connectivity among the top models inferred by GISE, suggesting that these connections likely are true positives.

(11) $$\begin{array}{l} \frac{d[E_{2}]}{dt}=p_{1}[E_{2}]+\frac{p_{2}}{p_{3}+[{OHE}_{2}^{4}]}+p_{4}[{OHE}_{2}^{4}],\\ \frac{d[{OHE}_{2}^{2}]}{dt}=\frac{p_{5}}{p_{6}+[Me{OHE}_{2}^{2}]}+p_{7}[{OHE}_{2}^{2}]+p_{8}[Me{OHE}_{2}^{4}],\\ \frac{d[{OHE}_{2}^{4}]}{dt}=\frac{p_{9}[{OHE}_{2}^{2}]}{p_{10}+[{OHE}_{2}^{2}]}+p_{11}[E_{2}]+p_{12}[{OHE}_{2}^{4}]+p_{13}[Me{OHE}_{2}^{4}],\\ \frac{d[Me{OHE}_{2}^{2}]}{dt}=p_{14}[E_{2}]+p_{15}[Me{OHE}_{2}^{2}],\\ \frac{d[Me{OHE}_{2}^{23}]}{dt}=p_{16}[Me{OHE}_{2}^{23}]+p_{17}[{OHE}_{2}^{4}]+\frac{p_{18}[Me{OHE}_{2}^{23}]}{p_{19}[Me{OHE}_{2}^{23}]},\\ \frac{d[Me{OHE}_{2}^{4}]}{dt}=p_{20}[Me{OHE}_{2}^{4}]+p_{21}[{OHE}_{2}^{4}], \end{array}$$

## 6. Discussion

In this study we introduced Grammatical Immune System Evolution (GISE) and applied it to the evolution of nonlinear dynamic Bayesian models (NDBMs) to automatically reverse engineer models from biologically motivated time-series simulations and from real experimental data from the estrogen metabolism pathway. Grammars allow one to incorporate domain-specific knowledge and thereby reduce the search space. Grammatical Evolution (GE) has these advantages, but in a particular real-world application GE’s performance showed no statistically significant difference between the performance of a simple Monte Carlo search, which is likely due to the destructive nature of mutation and crossover in GE. Motivated by this, we used the GISE formalism to create a non-destructive somatic mutation operator. This somatic hypermutation operator is based on the diversity-generating mechanism used in the human adaptive immune system. The purpose of this operator was to traverse the model search space efficiently while preserving evolutionarily fit model features.

We showed that GISE can routinely infer the correct NDBM from time series for three interacting variables by cycle 10 with a modest population size of only 50, and that GISE performs significantly better than a Monte Carlo search with the same population size and number of cycles (Sec. 5.1), thus demonstrating that GISE promotes learning by preserving useful model components. Our previous hybrid GE approach in Ref. [7] required much larger evolutionary population parameters to routinely identify a correct model. We found that GISE scaled well when applied to a more complex simulated data set (Sec. 5.2, Fig. 5). Despite being forced to fit data simulated from another formalism (S-system), the inferred operon models were still able to fit the the nonlinearities while also possessing the interpretability of S-system models. Beyond accurately tracking the time-series profiles, it is important to identify the correct causal connections between the time varying biomarkers. One of the top 3 GISE models predicted all of the connections in the simulated S-system model. It is quite possible the S-system formalism would also result in false positive and negative connections if used to fit data simulated from another formalism or from real data. Thus, an important caveat to time-series modeling is that one should use feedback with experiment whenever possible to eliminate false positive and negative model elements from the top models; one cannot simply rely on the model with the highest fitness. Note that this caveat applies to all dynamic-model reverse engineering algorithms, and not just GISE. Figure 6 shows that GISE is able to fit the larger S-system simulations even when corrupted by significant measurement noise due to the ability of the UKF to incorporate noise into the modeling process. In order to accelerate the search for correct models, we seeded the grammar with domain-specific knowledge in the form of regulatory elements that capture the intrinsic nonlinearity of biological time series data. In future studies, we will test the use of more general, recursive grammars.

All mathematical models are an approximation, but the gold standard test of whether the best model has been identified is through prediction and feedback with a validation experiment. In Sec. 5.3 GISE discovered a model (Eq. 11) that tracks a set of estrogen metabolism time series with high accuracy (Fig. 7). Comparison with a previous mathematical model [32] showed that GISE can automatically identify improved models from real data. From Figure 7 the most marked improvements were for MeOHE_2_^23^ (2-MeOHE_2_) for which the previous model approaches the wrong stead-state limit, and MeOHE_2_^23^ (2-OH-3-MeOHE_2_) for which the previous model does not fit the peak feature of the time series profile. The GISE model Equation 11 should be viewed as a starting point for model development and we anticipate that feedback with experiment will refine and extend the model inferred in this work. The mathematical model in Ref. [32] modeled three additional quantities whose data were not available for this study. However, in a future study we plan to incorporate this additional data and generate new predictions for feedback with experiment. Biological time series contain considerably more causal information than gene expression or protein abundance at a single time point, and bioinformatics tools such as GISE are needed to infer predictive models from these data. An accurate dynamic model can reveal insight into biological relationships and may act as an *in silico* experimental tool to generate testable hypotheses. Among these experiments will be to test the effect of intrinsic noise and small perturbations on the system. Thus, an important feature of GISE is the use of a Kalman Filter which accounts for noise when estimating system parameters.

As the mechanism for generating model diversity in GISE, we focused on grammatical somatic hyper-mutation, which is ideally suited for refining programs in the same way the biological immune system uses hypermutation to create high-affinity antibodies. V(D)J recombination is another immunogenetic mechanism for creating antibodies that exhibits a high degree of amino acid diversity. Our application needed only a small population size and number of cycles, but for higher-dimensional networks, GISE may benefit from the diversity-generating ability of V(D)J recombination. V(D)J recombination can be implemented in the same manner as IHM. The V(D)J process works by a recombinase that recognizes recombination signal sequences (RSSs) in the DNA sequence, which flank the coding elements. During model creation, the grammar would insert into the untranscribed program RSSs that contain semantic constraints, then during V(D)J recombination the grammar would act as the recombinase to join coding ends together in such a way that the function of the model is preserved.

## Supplementary Material

**Table S1:** The parameters of the three models in Table 1. The indices of the parameter array p[] are given in the first column. All parameters converged to the iteration tolerance of 0.0001.

Parameter index	Model 1	Model 2	Model 3
1	34.636	50.905	35.291
2	0.010	0.010	0.010
3	−26.569	−41.936	−27.157
4	−15.797	−20.448	−15.970
5	−24.326	−5.687	−11.064
6	6.293	12.752	11.233
7	14.735	−5.539	105.480
8	100.323	116.132	0.010
9	0.010	0.010	−105.999
10	−100.741	−116.943	48.489
11	44.006	53.480	−17.228
12	−16.797	−12.813	−34.849
13	−30.255	−44.416	−13.966
14	−15.294	−41.280	24.941
15	32.353	54.247	−10.743
16	−16.740	–	0.010
17	0.010	–	–

**Table S2:** The parameters of Equation 11 model of the estrogen metabolism time-series data. The indices of the parameter array p[] are given in the first column. All parameters have converged to the iteration tolerance of 0.0001.

Parameter Index	Estrogen metabolism model parameters
1	−0.467
2	0.047
3	0.938
4	0.115
5	1.037
6	1.150
7	−0.576
8	−0.426
9	−0.322
10	0.010
11	0.218
12	−0.337
13	0.205
14	0.011
15	−0.152
16	−0.927
17	0.086
18	0.045
19	0.010
20	−0.043
21	0.189

## Figures and Tables

**Table 1:** Analytical form of the top 3 GISE models predicted for the target coupled system simulated by Equation 10. Model predictions are given in Figure 5. GISE models are listed in order of goodness of fit. The models were constrained by the grammar to use an operon formalism. True positive and false positive connections in these models can be determined by comparison with Equation 10.

GISE Model 1
*dy*_1_/*dt* = *p*_1_*y*_3_/(*p*_2_ + *y*_3_) + *p*_3_*y*_1_ + *p*_4_*y*_5_
*dy*_2_/*dt* = *p*_5_*y*_2_ + *p*_6_*y*_1_ + *p*_7_*y*_4_
*dy*_3_/*dt* = *p*_8_*y*_3_/(*p*_9_ + *y*_3_) + *p*_10_*y*_3_
*dy*_4_/*dt* = *p*_11_*y*_3_ + *p*_12_*y*_5_ + *p*_13_*y*_4_
*dy*_5_/*dt* = *p*_14_*y*_5_ + *p*_15_*y*_3_ + *p*_16_*y*_1_/(*p*_17_ + *y*_1_)
**GISE Model 2**
*dy*_1_/*dt* = *p*_1_*y*_3_/(*p*_2_ + *y*_3_) + *p*_3_*y*_1_ + *p*_4_*y*_5_
*dy*_2_/*dt* = *p*_5_*y*_2_ + *p*_6_*y*_1_ + *p*_7_*y*_4_
*dy*_3_/*dt* = *p*_8_*y*_3_/(*p*_9_ + *y*_3_) + *p*_10_*y*_3_
*dy*_4_/*dt* = *p*_11_*y*_3_ + *p*_12_*y*_5_ + *p*_13_*y*_4_
*dy*_5_/*dt* = *p*_14_*y*_5_ + *p*_15_*y*_2_
**GISE Model 3**
*dy*_1_/*dt* = *p*_1_*y*_3_/(*p*_2_ + *y*_3_) + *p*_3_*y*_1_ + *p*_4_*y*_5_
*dy*_2_/*dt* = *p*_5_*y*_2_ + *p*_6_*y*_1_
*dy*_3_/*dt* = *p*_7_*y*_2_/(*p*_8_ + *y*_2_) + *p*_9_*y*_3_
*dy*_4_/*dt* = *p*_10_*y*_3_ + *p*_11_*y*_5_ + *p*_12_*y*_4_
*dy*_5_/*dt* = *p*_13_*y*_5_ + *p*_14_*y*_3_ + *p*_15_*y*_3_/(*p*_16_ + *y*_3_)
